# Are systematic reviews up-to-date at the time of publication?

**DOI:** 10.1186/2046-4053-2-36

**Published:** 2013-05-28

**Authors:** Elaine M Beller, Joyce Kee-Hsin Chen, Una Li-Hsiang Wang, Paul P Glasziou

**Affiliations:** 1 Centre for Research in Evidence-Based Practice, Faculty of Health Sciences and Medicine, Bond University, Gold Coast, QLD 4229, Australia; 2 Department of Nursing, Taipei Medical University, Wan Fang Hospital, 111, Section 3, Hsing-Long Road, Taipei 116, Taiwan; 3 Graduate Institute of Clinical Medical Sciences, College of Medicine, Chang Gung University, 259 Wen-Hwa 1st Road, Kwei-Shan, Taoyuan 333, Taiwan; 4 School of Nursing, College of Nursing, Taipei Medical University, 250 Wu-Hsing Street, Taipei 110, Taiwan; 5 Center for Evidence-Based Medicine, Taipei Medical University, 250 Wu-Hsing Street, Taipei 110, Taiwan; 6 College of Nursing, Chang Gung University of Science and Technology, 261 Wen-Hwa 1st Road, Kwei-Shan, Taoyuan 333, Taiwan

**Keywords:** Systematic reviews, Reporting guidance, Quality of reporting, Up-to-date, Information retrieval, Dissemination of results, Presentation and publication policy, Time factors

## Abstract

**Background:**

Systematic reviews provide a synthesis of evidence for practitioners, for clinical practice guideline developers, and for those designing and justifying primary research. Having an up-to-date and comprehensive review is therefore important. Our main objective was to determine the recency of systematic reviews at the time of their publication, as measured by the time from last search date to publication. We also wanted to study the time from search date to acceptance, and from acceptance to publication, and measure the proportion of systematic reviews with recorded information on search dates and information sources in the abstract and full text of the review.

**Methods:**

A descriptive analysis of published systematic reviews indexed in Medline in 2009, 2010 and 2011 by three reviewers, independently extracting data.

**Results:**

Of the 300 systematic reviews included, 271 (90%) provided the date of search in the full-text article, but only 141 (47%) stated this in the abstract. The median (standard error; minimum to maximum) survival time from last search to acceptance was 5.1 (0.58; 0 to 43.8) months (95% confidence interval = 3.9 to 6.2) and from last search to first publication time was 8.0 (0.35; 0 to 46.7) months (95% confidence interval = 7.3 to 8.7), respectively. Of the 300 reviews, 295 (98%) stated which databases had been searched, but only 181 (60%) stated the databases in the abstract. Most researchers searched three (35%) or four (21%) databases. The top-three most used databases were MEDLINE (79%), Cochrane library (76%), and EMBASE (64%).

**Conclusions:**

Being able to identify comprehensive, up-to-date reviews is important to clinicians, guideline groups, and those designing clinical trials. This study demonstrates that some reviews have a considerable delay between search and publication, but only 47% of systematic review abstracts stated the last search date and 60% stated the databases that had been searched. Improvements in the quality of abstracts of systematic reviews and ways to shorten the review and revision processes to make review publication more rapid are needed.

## Background

Systematic reviews provide a synthesis of evidence for practitioners, for clinical practice guideline developers, and for those designing and justifying new primary research [1,2]. Because systematic reviews help to set new trials in the context of previous similar research, some healthcare journals have made this a requirement for reporting new research [3]. An up-to-date systematic review should also be considered before future trials on the same topic are conducted [4]. Being able to readily identify up-to-date and comprehensive systemic reviews is therefore important to several groups. Hence the PRISMA Statement (Preferred Reporting Items for Systematic Reviews and Meta-analyses) guideline requires describing the information sources (item 7) and the search method (item 8) of systematic reviews [5,6]. These items suggest that review authors describe all information sources searched (for example, databases with dates of coverage, contact with study authors to identify additional studies, and the date they were last searched) [6]. The PRISMA Statement also suggests including the first of these (information sources) in the abstract of the systematic review report [5].

Many readers of systematic reviews scan only the abstract in order to determine the relevance of the review to their needs [7]. Part of this scan should assess the comprehensiveness and recency of the review. For users of reviews the crucial date for assessing recency is the date of last search, rather than the date of publication. Whilst more complex algorithms for assessing whether a review is up-to-date exist [8], the length of delay from last search is a simple way of assessing the recency of a review when scanning abstracts for relevant papers on a topic.

Although the search dates are usually reported in the main text of reviews, the reporting of these in abstracts is less well documented. We believe that the dates should be reported in the abstract, as this is often where readers assess whether to obtain the full text of articles [7].

Delays in publication are well documented for some types of research. For clinical trials, one study showed that the median time from completion to first submission of the main results was 10 months, and the time to publication was 23 months [9]. An analysis of 100 systematic reviews suggested that new research published between the conduct and publication of the review meant that 7% of the reviews were out of date on the day of publication, but did not analyze the length of delay between search and publication [8]. A study in 2008, prior to the release of the PRISMA Statement, found that the median time from search to publication was 61 weeks [10]. To document the extent of delay between search and publication in more recent systematic reviews, we decided to sample reviews published between 2009 and 2011 to determine the dates of search completion in relation to the date of publication, and how well this was reported in the abstract and full text of the review.

### Objectives

The primary study objective was to evaluate how up-to-date systematic reviews are at the time of first publication, as measured by the time lag from last search date to publication. Secondary objectives were to ascertain how much of the time from search date to publication was caused by delays in submission and revision of manuscripts, as compared with delays in the publishing lead time, and to determine whether authors provided information on search dates and database sources in the abstract of the review, as this is often the only part of a systematic review that is read by someone screening for relevant papers.

## Methods

### Data search, study selection and data extraction

We collected all systematic review articles indexed in Medline each year from 2009 to 2011 from the National Library of Medicine’s Core Clinical Journals (CCJ) subset of journals [11]. The CCJ subset was chosen because we wanted a broader selection of journals than the major general medical ones, but needed to limit the search due to the large number of citations to screen to determine which of these were systematic reviews. The CCJ journals are those ‘recommended for individual practitioners and libraries of small hospitals and clinics’ [11]. We used the broad definition of a systematic review previously used by Moher and colleagues in their study of the epidemiology of systematic reviews: ‘… the authors’ stated objective was to summarize evidence from multiple studies, and the article described explicit methods, regardless of the details provided’ [12]. The eligible reviews were found using the same search strategy as was used in their study. One reviewer screened titles and abstracts initially, and then full texts, to determine whether the article was a systematic review using only two criteria: that a search strategy was described, and it appeared that all eligible papers were used in the review (for example, table of included studies or similar). A second reviewer independently assessed any reports where the classification was deemed unclear. All systematic reviews about interventions (*n* = 860) formed the population from which to sample. Using the random number generator in Excel, we randomly selected 100 intervention reviews from each year.

Data were collected from abstracts and full texts by one reviewer (EMB), with a 10% sample also independently extracted by two reviewers (JK-HC and UL-HW) for quality checking. The data extraction items included the following descriptive information: name of the journal, first author, and year of publication. From each study we extracted details on date of search, date of first publication (for example, online publication if ahead of print), and date of acceptance (where available). If the exact search date was not presented, the end of the month was used (for example, 31 October). Additionally, we checked the date of publication from the journal website if it was not printed on the article. Finally, the databases that had been searched in each systematic review were recorded.

### Outcome measures

First, the primary outcome was measured using the time from the last search date to the first date of publication. Second, delays in submission and publication were measured using the time from the last search date to the date of acceptance (where available), and the time from acceptance to first publication. Finally, the proportion of articles reporting the last search date and data sources in the full text and abstract was calculated.

### Data management and statistical analysis

All data extraction was managed by Microsoft Office InfoPath. Statistical analysis was conducted with SPSS, v.17 (Chicago, IL, USA). Descriptive statistics were used to summarize the data, using the number and proportion (%) to describe categorical variables and the mean, median, minimum, maximum and standard deviation for continuous variables. A survival analysis was conducted to determine the median time from search to acceptance and publication in published systematic reviews. A Kaplan–Meier curve was used to represent graphically the results of the survival analysis.

## Results

### Publication location of systematic reviews

Systematic reviews of interventions appeared in 85 of the 118 CCJ journals during 2009 to 2011. Six journals had more than 10 reviews of interventions published in that period (*BMJ*, *Annals of Internal Medicine*, *British Journal of Surgery*, *Annals of Surgery*, *Pediatrics*, *Lancet*). Thirty-five of the journals published only one systematic review of interventions during that time.

### Time from search to publication

**Figure 1:**
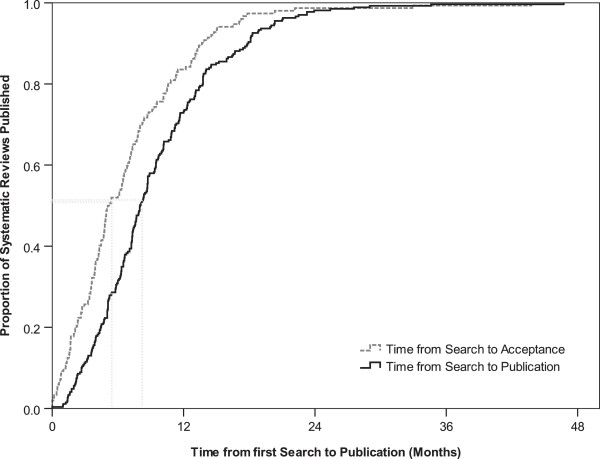
**Kaplan–Meier curve demonstrating the time to publication of 300 systematic reviews.** The median (minimum to maximum) time from last search to acceptance was 5.1 (0 to 43.8) months (95% confidence interval =3.9 to 6.2) and from last search to first publication time was 8.0 (0 to 46.7) months (95% confidence interval =7.3 to 8.7).

We included 300 systematic reviews. The median (minimum to maximum) time from last search to acceptance was 5.1 (0 to 43.8) months (95% confidence interval = 3.9 to 6.2) and from last search to first publication time was 8.0 (0 to 46.7) months (95% confidence interval =7.3 to 8.7), respectively. The times are shown with the Kaplan–Meier curve in Figure 1.

### Search date and databases stated in the abstract and full text

**Figure 2:**
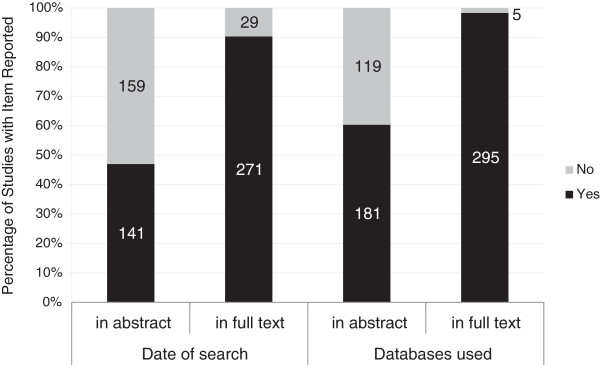
**Date of last search and databases searched stated in full text and abstract.** Percentage of systematic reviews with date of last search and databases searched stated in the full text and abstract.

In the full text of articles, 90.3% (271/300) stated the search date and 98.3% (295/300) stated the databases that were searched. However, only 47.0% (141/300) of articles stated the search dates and 60.3% (181/300) stated the databases that were searched in the abstract. Interestingly, there were respectively 29 (9.7%) and five (1.7%) articles that did not provide the search date and databases they used even in the full text, as shown in Figure 2.

### Characteristics of information sources in systematic reviews

**Figure 3:**
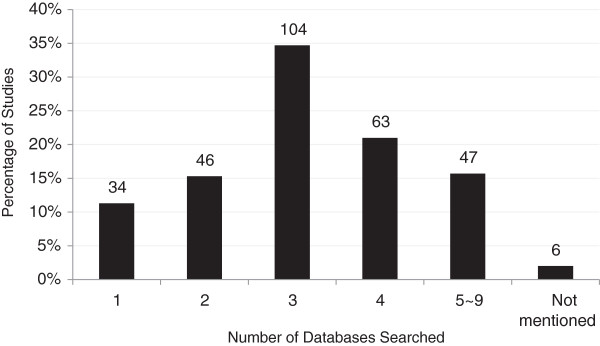
Number of databases searched in 300 published systematic reviews.

In 300 included systematic reviews, the mean (standard deviation) number of databases searched was 3.2 (1.6), with the range of databases being one to nine. Most researchers searched three (34.7%, 104/300) or four (21.0%, 63/300) databases in their systematic review. Thirty-four (11.3%) searches were conducted on only one database. Another six (2.0%) articles did not mention how many databases had been searched, as shown in Figure 3. Overall, the top three most used databases were MEDLINE (78.9% of reviews), Cochrane library (76.0%), and EMBASE (63.5%), as shown in Table 1.

**Table 1:** Databases searched in 300 systematic reviews

**Category**	**Name of database**	**Count (%)**
Critically-appraised databases	Cochrane library	228 (76.0%)
	DARE	14 (4.7%)
	CENTRAL	101 (33.7%)
	PEDro	7 (2.3%)
	Health Technology Assessment Database (HTA)	6 (2.0%)
Indexing and abstracting databases	MEDLINE	236 (78.9%)
	EMBASE	190 (63.5%)
	PubMed	86 (28.7%)
	CINAHL	52 (17.4%)
	PsycINFO	24 (8.1%)
	ERIC	12 (4.0%)
	LILACS	12 (4.0%)
	AMED Allied and Complementary Medicine	15 (5.0%)
	HealthSTAR	6 (2.0%)
	BIOSIS	6 (2.0%)
	Chinese/ China Biological Medicine Database	5 (1.7%)
Citation searching	Scopus	16 (5.4%)
	ISI Web of Science	8 (2.7%)
Trials registry	National Research Register	10 (3.3%)
	Clinicaltrials.gov	9 (3.0%)
	FDA Repository	3 (1.0%)
Online full-text journals	BioMed Central	4 (1.3%)
Web search	Google Scholar	8 (2.7%)
Hand searching	Conference proceedings	6 (2.0%)

Note: Other databases (*n* = 19, searched in <1% of reviews) included PROQUEST, International Pharmaceutical Abstracts, AEGIS, Popline and African Journals Online, Index for Australian Medical Literature, CBMdisc, Eastern Mediterranean Index, EBM Reviews, Health Economic Evaluations Database (HEED), European Society, ExtraMed, Imbiomed, Korean Studies Information Service System (KISS), Oxford Database of Perinatal Trials, Scholars Portal, York Centre for Reviews and Dissemination, International Pharmaceutical, and National Research Register.

## Discussion

Of the 90% of our 300 systematic reviews that provided a date of search, the median time from last search to publication was 8.0 months. This is an improvement over the results reported in 2008 where the median time was around 14 months [10]. However, the distribution in our study was skewed, with around 10% of reviews having a last search date to publication time of more than 18 months. Since reviews can date rapidly [8], this delay is important to users of reviewers.

For a reader searching for an up-to-date review, the relevant date is that of the last search not the date of publication, but this was provided in only 47% of abstracts. Hence readers would need to check, and possibly purchase, the full text to determine recency. Similarly, readers may wish to know the list of databases searched to assess completeness of the review, but this was missing from 40% of abstracts.

The time from search to publication can be usefully compared with the half-life of a review’s conclusions. One analysis of 100 systematic reviews found the half-life was 5.5 years until there was a change in the clinical conclusions of a review [8]. That analysis also found that 7% of reviews were out of date on the day of publication. That is, new research that changed the clinical conclusions was published between the date of search and the date of publication. This is consistent with our finding of a median time from last search to publication delay of 8.0 months.

We found no previous studies on the reporting of dates in abstracts, but several studies have examined the search dates and other items in the full text of reviews. An analysis of 65 Cochrane reviews found that 91% reported the years searched, but only 11% gave the date of last search [13]. Similarly, a study of 297 systematic reviews found that 70% reported the dates covered by the search, and 77% gave the end date of search, but these were better reported in Cochrane reviews (83% and 91%, respectively) than in non-Cochrane reviews (60% and 67%, respectively) [14].

Our analysis has some limitations. First, we only selected systematic reviews from MEDLINE's CCJ. If the noncore journals have longer delays then our results are likely to be an underestimate for all reviews. Second, we could only analyze the time from acceptance to publication, and only in some of the reviews. It would be helpful to obtain data on other components such as time for review, revision, re-review, and how often authors did search updates during the revision process. Third when authors did not present an exact date of search we rounded up to the end of the month (for example, October was coded as 31 October).

Given clinicians’ and other decision-makers’ needs for up-to-date reviews, the current length of delay and lack of dates in abstracts needs improvement. Journal publishers need to work with authors to find ways to shorten the time between search and publication. This could be through more rapid review and revision processes, or by providing means to do an additional prepublication search, as some Cochrane review groups do. Authors and editors should both ensure that the date of last search is included in the abstract, in keeping with the PRISMA Statement guidance. Editors and peer reviewers should expect authors to demonstrate compliance with the PRISMA Statement guidance on submission of their article. Publication of the search date in the abstract would make future monitoring of publication delays more feasible.

## Conclusions

Being able to identify comprehensive, up-to-date reviews is important to clinicians, guideline groups, and those designing clinical trials. This study demonstrates that some reviews have a considerable delay between search and publication; only 47% of systematic reviews abstracts stated the last search date; and 60% stated the databases that had been searched. To aid readers in rapidly determining the recency of a systematic review, we believe that the date of search should be present in its abstract. Improvements in the quality of abstracts of systematic reviews and ways to shorten the review and revision processes to make review publication more rapid are needed.

## Abbreviations

CCJ: Core Clinical Journals; PRISMA: Preferred Reporting Items for Systematic Reviews and meta-analyses.

## Competing interests

All authors declare that they have no competing interests.

## Authors’ contributions

PPG and EMB conceived the idea for the study and design the research protocol. EMB devised the search strategy, searched for studies, and extracted data from abstracts and full texts into Microsoft Office InfoPath. JK-HC and UL-HW extracted 10% data in duplicate for quality checking. JK-HC, UL-HW and EMB conducted the statistical analysis. All authors contributed to data interpretation. JK-HC and UL-HW drafted the manuscript. All authors contributed to revision of the manuscript and approved the final version. All authors read and approved the final manuscript.
